# Ischemic stroke caused by large-artery atherosclerosis: a red flag for subclinical coronary artery disease

**DOI:** 10.3389/fneur.2023.1082275

**Published:** 2023-04-12

**Authors:** Ana Luíza Vieira de Araújo, Raul D. Santos, Marcio Sommer Bittencourt, Roberto Nery Dantas, Carlos André Oshiro, Cesar Higa Nomura, Edson Bor-Seng-Shu, Marcelo de Lima Oliveira, Claudia da Costa Leite, Maria da Graça Morais Martin, Maramelia Miranda Alves, Gisele Sampaio Silva, Victor Marinho Silva, Adriana Bastos Conforto

**Affiliations:** ^1^Hospital das Clinicas, Neurology Clinical Division, University of São Paulo, São Paulo, Brazil; ^2^Heart Institute (Instituto do Coração), University of São Paulo, Medical School Hospital, São Paulo, Brazil; ^3^Hospital Israelita Albert Einstein, Academic Research Organization, São Paulo, Brazil; ^4^Division of Cardiology, Department of Medicine, University of Pittsburgh, Pittsburgh, PA, United States; ^5^Department of Radiology and Oncology, University of São Paulo, São Paulo, Brazil; ^6^Neurology and Neurosurgery Department, Universidade Federal de São Paulo (UNIFESP), São Paulo, Brazil

**Keywords:** ischemic stroke, coronary calcium score, subclinical coronary artery disease, coronary atherosclerosis, cervicocephalic atherosclerosis

## Abstract

**Background:**

The coronary calcium score (CAC) measured on chest computerized tomography is a risk marker of cardiac events and mortality. We compared CAC scores in two multiethnic groups without symptomatic coronary artery disease: subjects in the chronic phase after stroke or transient ischemic attack and at least one symptomatic stenosis ≥50% in the carotid or vertebrobasilar territories (Group_athero_) and a control group (Group_control_).

**Methods:**

In this cross-sectional study, Group_athero_ included two subgroups: Group_ExtraorIntra_, with stenoses in either cervical *or* intracranial arteries, and Group_Extra&Intra_, with stenoses in at least one cervical and one intracranial artery. Group_control_ had no history of prior stroke/transient ischemic attacks and no stenoses ≥50% in cervical or intracranial arteries. Age and sex were comparable in all groups. Frequencies of CAC ≥100 and CAC > 0 were compared between Group_athero_ and Group_control_, as well as between Group_ExtraorIntr_, Group_Extra&Intra_, and Group_control_, with bivariate logistic regressions. Multivariate analyses were also performed.

**Results:**

A total of 120 patients were included: 80 in Group_athero_ and 40 in Group_control._ CAC >0 was significantly more frequent in Group_athero_ (85%) than Group_control_ (OR, 4.19; 1.74–10.07; *p* = 0.001). Rates of CAC ≥100 were not significantly different between Group_athero_ and Group_control_ but were significantly greater in Group_Extra&Intra_ (*n* = 13) when compared to Group_control_ (OR 4.67; 1.21–18.04; *p* = 0.025). In multivariate-adjusted analyses, “Group_athero_” and “Group_Extra&Intra_” were significantly associated with CAC.

**Conclusion:**

The frequency of coronary calcification was higher in subjects with stroke caused by large-artery atherosclerosis than in controls.

## Introduction

Unlike myocardial infarction which is caused by atherosclerosis in more than 90% of the cases (1), only ~25% of ischemic strokes (IS) are attributable to atherosclerosis (2–4). Classification systems based on results of clinical, neuroimaging, and laboratory tests aim to determine the most likely stroke etiology. According to TOAST (Trial of Org 10172 in Acute Stroke Treatment) criteria, a diagnosis of “large-artery atherosclerosis” can be made in the presence of clinical and brain imaging findings consistent with >50% stenosis or occlusion of a major brain artery or a branch cortical artery, presumably due to atherosclerosis (5). A diagnosis of “evident large-artery atherosclerosis” can be made according to the Causative Classification of Stroke System (CCS), if the severity of the stenosis is ≥50% in intracranial or cervical arteries that supply the territory affected by the stroke, and other causes are excluded (6).

Patients with IS may have polyvascular disease with concomitant coronary (20%) or peripheral artery disease (22%) (7–9). Two seminal studies assessed the rates of asymptomatic coronary artery disease (CAD) in patients with IS or transient ischemic attack (TIA) in countries with predominantly White populations. Rokey et al. prospectively assessed patients with mild IS or TIA with Thallium-201 scintigraphy and exercise radionuclide ventriculography (10). Abnormal cardiac scans consistent with CAD were found in 41.2% (14/34) of the patients with no history or ECG signs of myocardial ischemia. Chimowitz et al. (11) specifically addressed the rates of asymptomatic CAD in patients with cerebrovascular ischemic disease of different etiologies. Abnormal stress tests were significantly more frequent (50%; 15/30) in patients with large-artery IS or TIA and no symptoms of CAD than in patients with other causes of brain ischemia (23%; 9/39). In studies performed more than a decade later, asymptomatic coronary artery stenosis ≥50% was described in 18–20% of French patients with non-cardioembolic IS and associated with an increased risk of death (12, 13).

The coronary calcium score (CAC) quantified on chest non-contrast computed tomography (CT) is an excellent non-invasive predictor of atherosclerotic cardiovascular risk (14–16). CAC is a surrogate of coronary plaque burden and is independently associated with the risk of myocardial infarction or mortality (17–19). In the MESA study, the annual frequencies of cardiovascular events in asymptomatic subjects were: CAC = 0, 0.4%; CAC 1–99, 0.8%; and CAC ≥ 100, 2.4% (20).

In the MESA study, CAC = 0 identified a group of individuals with a very low risk of events in 11 years of follow-up. CAC > 0 encompasses all positive scores and significantly identifies individuals with a greater risk of events in comparison with those with CAC = 0. CAC ≥ 100 has been classically used to make comparisons with CAC = 0 and is a marker of atherosclerotic cardiovascular disease (ASCVD) events consistent with the recommendation of the ACC/AHA guidelines of ≥0.75% per year risk of myocardial infarction, death, and stroke-a value considered as a threshold to justify the use of statins in primary prevention for people without overt hypercholesterolemia (21).

In Japanese patients with IS not caused by cardiac embolism or symptomatic carotid artery disease, without symptoms of CAD, absolute CAC scores were significantly higher than in controls, suggesting a greater risk of MI or death (22). In Korean patients with IS, without symptoms of CAD, CAC scores were associated with severe CAD assessed by computed tomography coronary angiography. Severe CAD was also associated with the presence of stenoses due to atherosclerosis in cervicocephalic arteries (23). In Chinese patients, CAC scores were significantly higher in patients with acute IS caused by atherosclerosis than in a control group composed of subjects with asymptomatic carotid atherosclerosis (24).

There is limited information about subclinical coronary artery disease in multiethnic patients with ischemic stroke caused by large-artery atherosclerosis. The main goal of this study was to compare CAC scores in subjects with IS specifically caused by large-artery atherosclerosis (Group_athero_) and in controls (Group_control_) in Brazil, a country with a highly miscegenated population. We hypothesized that the frequency of CAC ≥100 and CAC >0, as well as absolute CAC scores, would be higher in Group_athero_ than in Group_control_. In addition, we expected that patients with symptomatic cervical and intracranial stenoses ≥50% due to atherosclerosis (Group_Extra&Intra_) would have a greater extent of subclinical CAD than patients with symptomatic, exclusively cervical or intracranial stenoses ≥50% (Group_ExtraorIntra_).

## Methods

### Study design and participants

In this cross-sectional study, patients were recruited from two outpatient stroke clinics at public university hospitals (Hospital das Clínicas/São Paulo University and São Paulo Hospital/São Paulo Federal University) in Brazil between September 2015 and March 2018. Controls with comparable age and sex distributions were recruited from non-consanguineous companions of patients in order to include subjects with similar socioeconomic status and access to healthcare in a large urban center of a middle-income country. Age and sex of the included patients were continuously updated in the enrollment log. A subject would be invited to participate in Group_control_ if he/her had the same sex of a patient and his/her age matched the age of the patient ± 10 years. The protocol was approved by the Institutional Review Board (protocol number 1.175.113), and all patients provided written informed consent.

### Eligibility criteria

Subjects aged 45–80 years were included. History/symptoms of coronary heart disease or pathologic Q waves in the electrocardiogram were exclusion criteria for both groups. Electrocardiograms were performed in all subjects prior to inclusion. Specific criteria for patients with atherosclerosis *(*Group_athero_) and controls (Group_control_) are listed below.

#### Atherosclerosis group (Group_athero_)

Inclusion criteria: The inclusion criteria were as follows: IS in the internal carotid artery or vertebrobasilar territory in the past 15 years, confirmed by computerized tomography (CT) or magnetic resonance imaging; stenosis ≥50% in cervical, intracranial, or both segments of these arteries, diagnosed by computed tomography angiography, magnetic resonance angiography (MRA), or digital subtraction angiography within 6 months post-stroke.

Exclusion criterion: IS etiology different from evident or probable large-artery atherosclerosis, according to the CCS (5, 21).

Group_athero_ was divided into two subgroups: Group_ExtraorIntra_ (stenoses ≥50% in either a cervical or an intracranial artery supplying the territory affected by IS) and Group_Extra&Intra_ (stenoses ≥50% in at least one cervical and at least one intracranial artery).

#### Controls (Group_control_)

Inclusion criteria were age and sex comparable to those of subjects in Group_athero_. Exclusion criteria were a history of transient ischemic attack (TIA) or stroke; stenoses ≥50% in a cervical or intracranial artery diagnosed by MRA or transcranial Doppler and cervical Doppler. Cervical and intracranial MRA or cervical and transcranial Doppler were performed in all subjects.

### Characteristics of the subjects

Demographic data, history of hypertension, diabetes, hypercholesterolemia, peripheral artery disease, smoking, and metabolic syndrome were assessed on the day of inclusion in the study. Definitions are shown in Supplementary material 1. The use of antihypertensive, antidiabetic, antiplatelet drugs, or statins was also registered. The results of routine laboratory exams from Group_athero_ were retrieved from electronic records. Tests were ordered for controls and patients if no blood workup had been performed within 6 months prior to enrollment.

Cardiovascular risk was estimated by the *pooled cohort equations (PCE*), a well-established, global measure of vascular risk according to the 2013 ACC/AHA recommendations, assessed according to the following variables: sex, age, race, total cholesterol, HDL cholesterol, systolic blood pressure, diabetes, and smoking status (25). This quantitative risk assessment method predicts the 10-year risk of developing a first cardiovascular event, defined as non-fatal myocardial infarction, death from CAD, or fatal or non-fatal stroke among people with no cardiovascular disease (26, 27).

The severity of neurological impairments caused by stroke was defined by scores in the National Institutes of Health Stroke Scale (NIHSS) (28–30) and the severity of disability was evaluated by the modified Rankin Scale (29). Both scales were evaluated on the day of inclusion in the study.

### Outcomes

The primary outcome was CAC ≥100 in the two main groups (Group_athero_ and Group_control_). The secondary outcomes were CAC >0 and CAC absolute values in the main groups and CAC ≥100, CAC >0, and absolute CAC values in subgroups (Group_ExtraorIntra_ and Group_Extra&Intra_). Outcome analyses were adjusted for PCE scores.

#### Coronary calcium score

Coronary calcium score was acquired by a 320-detector row CT scanner (Aquilion ONE, Canon Medical System Corporation, Otawara, Japan) at the Heart Institute (InCor)/University of São Paulo Medical School, São Paulo, Brazil.

The protocol consisted of a prospective acquisition in the inspiratory apnea, under electrocardiographic gating with a tube voltage of 120 kV, and the current was adjusted according to the patient's body mass index. The collimation pattern of the apparatus was 320 × 0.5 mm and the rotation speed time was 0.35 s. Sequential slices with 3.0 mm spacing were obtained, which is the standard method in clinical practice, as previously described (17). The effective radiation dose (in mSv) was calculated and controlled in all cases.

#### CT image analysis

The images were fully analyzed through a dedicated workstation (Aquarius, Intuition Edition, TeraRecon Inc., Version 4.4.11, California, USA) by a single experienced cardiologist (RD) blinded to clinical data using the scoring system previously described by Agatston et al. (17). All subjects were categorized in CAC ≥100 or < 100, as well as in CAC = 0 or >0.

#### Statistical analysis

Continuous variables are expressed as mean ± standard deviation (SD), whereas categorical variables are presented as frequencies. Between-group comparisons of baseline characteristics were performed with unpaired *t*-tests, Mann–Whitney tests, likelihood tests, chi-square tests, or Fisher's exact tests, according to the nature and distribution of the data.

Frequencies of CAC = 0 or >0 and CAC < 100 or ≥100 between Group_athero_ and Group_control_, as well as between subgroups Group_ExtraorIntra_ or Group_Extra&Intra_ and Group_control_, were compared with bivariate logistic regression. Odds ratios (ORs) and 95% confidence intervals (95% CIs) were calculated. The sample size was not formally estimated because no preliminary data were available.

Multiple logistic regression was performed to identify independently associated factors of CAC ≥100 or CAC >0. In Model 1, the independent variables were PCE and Group_athero_ (Model 1).

In addition, in Model 2, we calculated “PCE_withoutstatinuse_” for statin users by estimating the likely LDL-C level in the absence of statin use as previously described [LDL-C level+(30% x LDL-C level)] (31). This analysis was performed because there is evidence that statin therapy may influence CAC (32). The independent variables were PCE_withoutstatinuse_ and Group_athero_.

Comparisons of absolute CAC values between groups were performed with the Mann–Whitney test and between Group_ExtraorIntra_, Group_Extra&Intra_, and Group_control_, with the Kruskal–Wallis test. *Post-hoc* analyses were made with Dunn's multiple comparisons.

We also evaluated absolute calcium scores as a continuous variable, using the base-10 logarithm of the sum of the coronary calcium score plus 1 [log10 (CAC + 1)]. The addition of 1 to the calcium score before logarithmic transformation was performed so that patients with a calcium score of zero could be included in the analysis as previously described.

A *p*-value of < 0.05 was considered statistically significant. The tests were performed using SPSS for Windows version 22.0.

## Results

### Characteristics of the subjects

**Figure 1:**
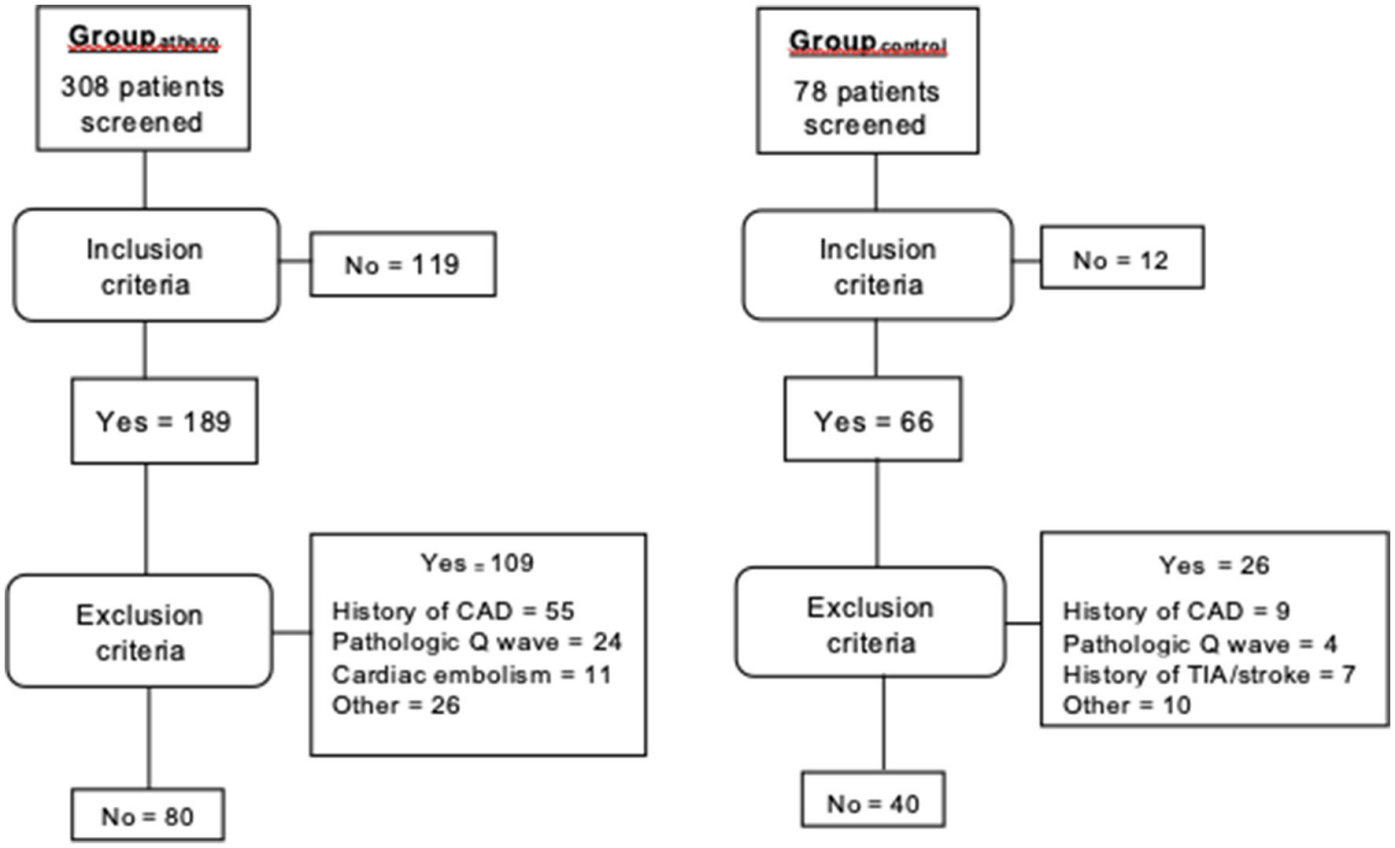
Flow diagram. CAD, coronary artery disease; TIA, transient ischemic attack. Some subjects had more than one exclusion criteria.

Figure 1 shows the flowchart of inclusion. Table 1 shows the baseline characteristics of the subjects in Group_athero_ (*n* = 80) and Group_control_ (*n* = 40). In Group_athero_, the median modified Rankin score was 2 (IQR, 2); the median NIHSS at the time of inclusion was 1.5 (IQR, 3.3), and the median time from stroke onset was 2 years (0–11.5). More than half (55%) of the patients were assessed within the 1st-year post-stroke and 32.5% within 2–5 years.

**Table 1:** Characteristics of the subjects.

**Characteristic**	**Group_athero_ (*n* = 80)**	**Group_control_ (*n* = 40)**	***p*-value**
Age (years)	64.5 ± 7.6	64.2 ± 6.3	0.817^b^
Education (years)	6.8 ± 4.7	6.8 ± 4.9	0.766^c^
Male sex (%)	67.5	55	0.180^a^
**Ethnic group (%)**			
Black	44.4	47.5	0.302^d^
White	53.8	50	
Asian	3.8	2.5	
Hypertension (%)	87.5	75	0.083^a^
Diabetes (%)	45	42.5	0.795^a^
Hyperlipidemia (%)	100	70	**< 0.001** ^**e**^
Family history of stroke (%)	51.2	20	**0.001** ^**a**^
Pooled cohort equations risk (%)	20.2 ± 16.3	22.1 ± 15.3	0.794^b^
Smoking (%)	15	12.5	0.923^a^
Ankle-brachial Index < 0.9 (%)	29	5	**0.002** ^**a**^
Metabolic syndrome (%)	47.5	50	0.796^a^
Antiplatelet agents (%)	92.5	17.5	**< 0.001** ^**a**^
Statins (%)	97.5	40	**< 0.001** ^**a**^
Anti-diabetic medications (%)	28.7	12.5	**0.047** ^**a**^

Means ± standard deviations are given.

^a^Chi-square test.

^b^Student's t-test.

^c^Mann–Whitney test.

^d^Likelihood test.

^e^Fisher's test.

*p* values < 0.05 were highlighted in bold.

There were no significant differences between groups in relation to age, sex, rates of hypertension, diabetes mellitus, smoking, metabolic syndrome, or in the estimated cardiovascular risk according to PCE. Hyperlipidemia, family history of stroke, abnormal ankle-brachial index, use of antiplatelet drugs, statins, and antidiabetic and antihypertensive drugs were more frequent in Group_athero_ than in Group_control_.

There were no significant differences in characteristics between subjects in Group_Extra&Intra_ and in Group_ExtraorIntra_ (Supplementary Table 1).

## Outcomes

### Primary outcome: CAC ≥100 in main groups

CAC ≥100 was present in 46.3% (*n* = 37) subjects in Group_athero_ and 32.5% (*n* = 13) in Group_control_ (OR, 1.79; 95% CI, 0.81–3.96; *p* = 0.152). Table 2 shows the results of univariate subgroup analyses. CAC ≥100 was significantly more frequent in Group_Extra&Intra_ than in patients in Group_control_. There were no differences between proportions of CAC ≥100 in Group_Extra&Intra_ and in Group_ExtraorIntra_.

**Table 2:** Comparisons in rates of coronary calcium scores (CAC) ≥100 or >0 between Group_control_, Group_ExtraorIntra_, or Group_Extra&Intra_.

**Subgroups**	**CAC ≥100, *n* (%)**	**OR (95% CI)**	***p*-value**	**CAC > 0, *n* (%)**	**OR (95% CI)**	***p*-value**
Group_control_	13 (32.5)	1.00		23 (57.5)	1.00	
Group_ExtraorIntra_	28 (41.8)	1.49 (0.66–3.39)	0.34	56 (83.6)	3.76 (1.53–9.26)	0.004
Group_Extra&Intra_	9 (69.2)	4.67 (1.21–18.04)	**0.025**	12 (92.3)	8.87 (1.05–74.95)	**0.045**

OR, odds ratio; CI, confidence interval; CAC, coronary artery calcification scores; OR calculated using bivariate logistic regression.

*p* values < 0.05 were highlighted in bold.

### Secondary outcomes

#### CAC >0 in main groups and in subgroups

CAC >0 was found in 85% (*n* = 68) subjects in Group_athero_ and 57.5% (*n* = 23) in Group_control_ (OR, 4.19; 95% CI, 1.74–10.07; *p* = 0.001). Table 2 shows the results of subgroup analyses. CAC >0 was significantly more frequent in Group_ExtraorIntra_ or Group_Extra&Intra_ than in Group_control_.

#### Absolute CAC values in main groups and in subgroups

CAC scores were significantly higher in Group_athero_ (median, 75.4; range: 0–2766.1) compared to Group_control_ (median, 11.7; range: 0–2153.7) (*p* = 0.024).

CAC absolute values were significantly greater in Group_Extra&Intra_ (median 109.51; range: 0–2766) and Group_ExtraorIntra_ (median 56.26; range: 0–1817) than in Group_control_ (*p* = 0.028), but the *post-hoc* analysis did not show significant differences between Group_ExtraorIntra_ and Group_control_ (*p* = 0.194), Group_Extra&Intra_ and Group_control_ (*p* = 0.075), or Group_ExtraorIntra_ compared to Group_Extra&Intra_ (*p* = 0.308).

#### Independent factors associated with CAC ≥100, CAC >0, and CAC absolute values

In multiple logistic regression, the variable “Group_athero_” was significantly associated with CAC >0 and Log (CAC + 1) (Table 3).

**Table 3:** Multivariate analyses.

**Model 1**	**OR (95% CI)**	***p*-value**
**CAC** ≥**100**^a^		
PCE	1.026 (1.002–1.052)	**0.035**
Group_athero_	1.769 (0.787–3.975)	0.167
**CAC** > **0**^b^		
PCE	1.026 (0.994–1.06)	0.108
Group_athero_	4.229 (1.735–10.305)	**0.002**
**Log (CAC** + **1)**^c^		
PCE	0.039 (0.012–0.066)	**0.005**
Group_athero_	1.021 (0.097–1.945)	**0.030**

^a^Multiple logistic regression: dependent variables, presence of coronary calcium scores (CAC) ≥100; Independent variables, scores in pooled cohort equations and group (Group_*athero*_ or Group_*control*_).

^b^Multiple logistic regression: dependent variables, presence of coronary calcium scores (CAC) ≥0 Independent variables, scores in pooled cohort equations and group (Group_*athero*_ or Group_*control*_).

^c^Linear regression: dependent variable, logarithm of sum (absolute coronary calcium scores + 1). Independent variables, scores in pooled cohort equations and group (Group_*athero*_ or Group_*control*_).

OR, odds ratio; CI, confidence interval; CAC, coronary artery calcification scores; PCE, pooled cohort equations.

*p* values < 0.05 were highlighted in bold.

**Figure 2:**
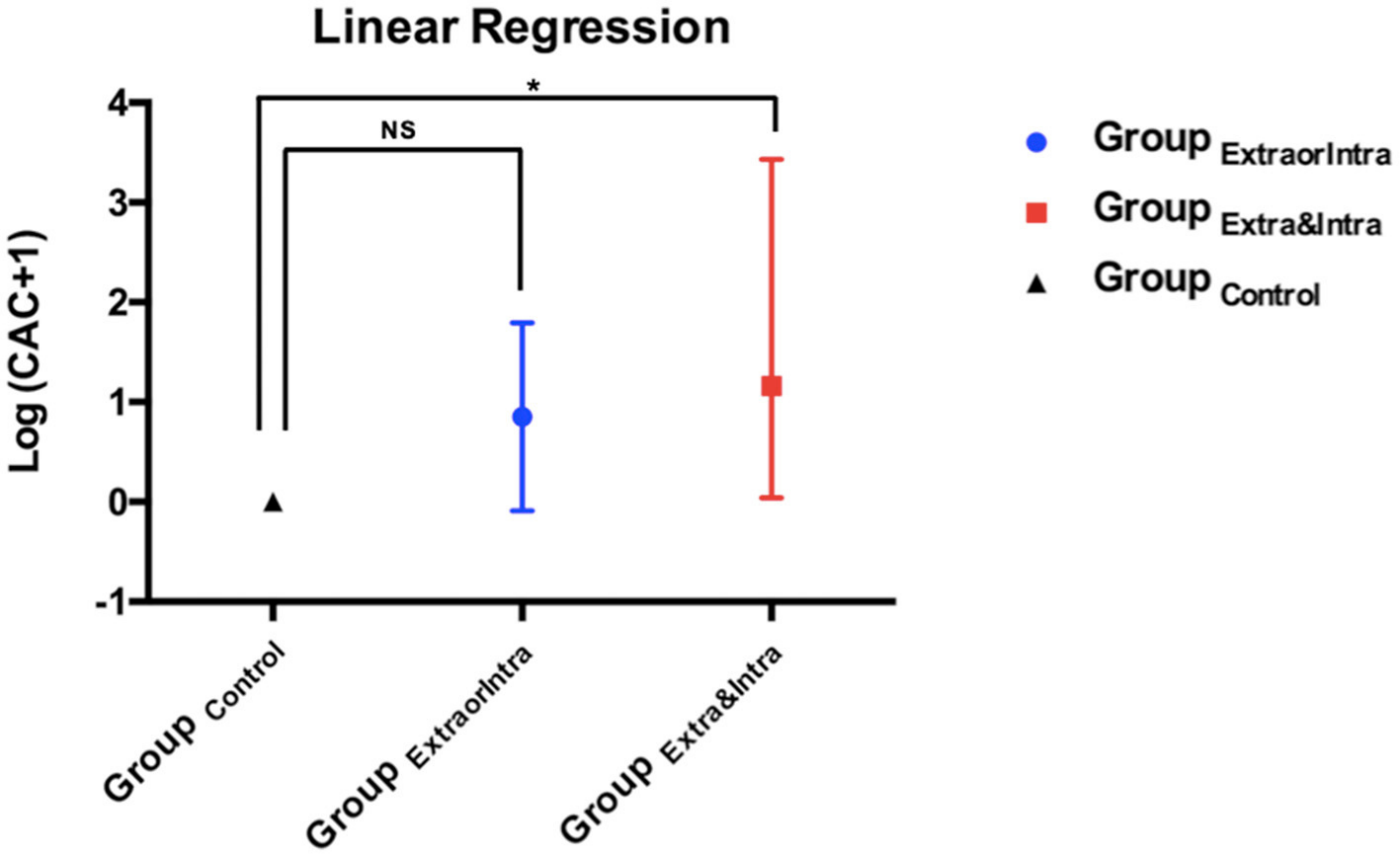
Linear regression of log (CAC + 1) between subgroups (Group_control_, Group_ExtraorIntra_, Group_Extra&Intra_). The asterisk indicates the statistically significant difference in reference to the control group. NS, non-statistically significant difference.

Figure 2 shows subgroup analyses of CAC absolute values. Only Group_Extra&Intra_ was significantly associated with Log (CAC + 1) (95% CI, 0.40–3.43; *p* = 0.013).

The results of Model 2 are shown in Supplementary Table 2. The results of multivariate analyses, with calculated “PCE_withoutstatinuse_” for statin users, were similar to those obtained in Model 1.

## Discussion

The main finding of this study was a significantly greater burden of subclinical coronary atherosclerosis in individuals with IS caused by cervicocephalic atherosclerosis than in controls. In Group_athero_, 85% of the patients had CAC >0 despite the absence of CAD symptoms. After adjusted analysis, stroke due to large-artery atherosclerosis was significantly associated with CAC >0 or CAC scores in comparison with controls.

The frequency of more extensive CAC (CAC ≥100) was higher in Group_athero_ than in Group_control_, but this difference was not statistically significant. Interestingly, CAC ≥100 was significantly more frequent in the subgroup with the biggest load of atherosclerosis (Group_Extra&Intra_) than in the subgroup with atherosclerosis restricted to intra- or extracranial arteries (Group_ExtraorIntra_). Multivariate analysis also showed a statistically significant association between Group_Extra&Intra_ and higher absolute levels of CAC, implying that patients with more extensive cervicocephalic atherosclerosis may be at a greater risk of subclinical coronary atherosclerosis and therefore a greater future risk of coronary events, than those with either cervical or intracranial atherosclerosis. This greater risk could point to a need for a more detailed assessment of CAD in these patients, especially considering that many subjects with stroke have physical disabilities that could mask the onset of angina symptoms related to mobility and, therefore, delay the diagnosis of obstructive CAD (33, 34). In addition, these results pave the way for future studies to investigate the effects of more aggressive treatment measures for this very high-risk subgroup, such as the use of PCSK9 inhibitors (35–37). Future clinical trials are needed to confirm this hypothesis.

Ethnicity may influence the distribution of atherosclerotic plaques across vascular beds. Intracranial atherosclerosis, for instance, predominates in Black, Hispanic, and Asian populations (38). CAC scores are higher in Black and Hispanic subjects than in White and Asian individuals (39). In our study, 44.4% of the patients were Black. Our results are in line with those of prior studies that investigated rates of subclinical CAD in patients from countries with predominantly White or Asian populations and IS caused by diverse etiologies (40, 41), non-cardioembolic stroke (12, 13), or atherosclerosis (24).

Patients with IS caused by atherosclerosis have a greater risk of cardiovascular events or all-cause mortality than controls with comparable estimated vascular risk (8). Antiplatelet drugs, statins, and other medications as well as behavioral interventions to control vascular risk factors are recommended for patients with IS caused by atherosclerosis according to current guidelines. However, adherence to secondary prevention measures may be challenging in clinical practice. Awareness of their great risk of cardiovascular death may strengthen the drive for patients to optimize compliance with medical therapy and changes in lifestyle.

CAC scores are associated with the risk of CAD and stroke in asymptomatic subjects (42, 43). In the present study, despite the lack of symptoms and the absence of stenoses ≥50% in cervical or intracranial arteries due to atherosclerosis, subjects in the control group were found to be at high cardiovascular risk according to PCE scores. Despite their high-risk profile, subjects in Group_control_ were significantly less likely to use medications to treat hypertension, diabetes, or dyslipidemia. This finding may reflect the underdiagnosis and treatment of these conditions in asymptomatic subjects in low- and middle-income countries like Brazil (44). Unfortunately, measures to control risk factors for vascular disease may only start after a major cardiovascular event such as stroke.

This study has some limitations. It has limited power for the comparison of rates of CAC ≥100 between Group_athero_ and controls. A multicenter study would be advisable to include a greater number of subjects for this comparison. The control group included subjects without cerebrovascular disease or stenoses ≥50% in cervical or intracranial arteries. Other studies are necessary to compare CAC scores in subjects with IS caused by large-artery atherosclerosis and in those with other IS etiologies. Moreover, the inclusion of time from a stroke in Group_athero_, up to 15 years, might lead to bias. Over the years, there might be a progression of coronary calcification as well as the worsening of control of cardiovascular risk factors. However, it is unlikely that this may have influenced our results because: First, more than half of the patients were assessed within the 1st year and < 15% more than 5 years post-stroke. Second, PCE scores were comparable between subjects with IS and controls. Age, a variable that substantially influences CAC scores (45), was also comparable between the groups. Third, the use of medications to control risk factors was found to be greater in the stroke group than in the control group. This could make the finding of greater CAC scores in the stroke group, compared to controls, less likely. Despite this, we found that the “stroke status” was an independent predictor of CAC >0 and, hence, greater cardiovascular risk. Fourth, multivariate analysis (Model 2), with “PCE_withoutstatinuse_” for statin users (estimation of the likely LDL-C level in the absence of statin use), showed the same results compared to Model 1, in which the independent variables were PCE and Group_athero_.

## Conclusion

The frequency of coronary calcification was higher in subjects with stroke caused by large-artery atherosclerosis than in controls. Stroke caused by large-artery atherosclerosis should be considered a red flag for subclinical coronary atherosclerosis, particularly in subjects with stenoses >50% in cervical and intracranial arteries.

## Data availability statement

The raw data supporting the conclusions of this article will be made available by the authors, without undue reservation.

## Ethics statement

The studies involving human participants were reviewed and approved by Comissão de Ética para Análise de Projetos de Pesquisa—CAPPesq—Hospital das Clínicas/São Paulo University. The patients/participants provided their written informed consent to participate in this study.

## Author contributions

AA and AC contributed to the concept, design, analysis, interpretation of data for the article, and drafted the manuscript. RS, MB, CN, EB-S-S, RD, MM, MA, GS, VS, and CO contributed to the acquisition, analysis, and interpretation of data for the work. RS, MB, CL, and GS critically revised the manuscript. All authors gave final approval and agree to be accountable for all aspects of work ensuring integrity and accuracy.
