# Supplementary material for: Ischemic stroke caused by large-artery atherosclerosis: a red flag for subclinical coronary artery disease
Source: Front Neurol. 2023 Apr 12;14:1082275. doi: 10.3389/fneur.2023.1082275 (PMC10130387; doi:10.3389/fneur.2023.1082275)
Supplement: Supplementary file 1 [file Data_Sheet_1.docx]

**Supplementary Material**

**Supplementary Document 1.** Definitions of variables.

1. Hypertension: reported in the medical record or current use of antihypertensive, or physical examination, according to guidelines of the Brazilian Society of Cardiology. (http://departamentos.cardiol.br/dha/vidiretriz/06-cap02.pdf)
2. Diabetes mellitus or fasting hyperglycemia: reported in the medical record or current use of medications to control blood glucose or altered fasting glucose tests or two-hour tolerance test. (http://www.diabetes.org.br/images/2015/area-restrita/diretrizes-sbd-2015.pdf)
3. Dyslipidemia: diagnosis and current treatment or result of total cholesterol and fractions. (http://publicacoes.cardiol.br/consenso/2013/V_Diretriz_Brasileira_de_Dislipidemias.pdf)
4. Smoking: information written on the medical record or referred by the patient. Note how many packs / year (packs / day x years of smoking, remembering that 1 pack has 20 cigarettes).

E) Family history of stroke or coronary artery disease: written on the patient record or referred to by the patient.

F) Ankle / brachial index: quotient between the highest ankle systolic pressure and the brachial systolic pressure. The ankle / brachial index below 0.90 is indicative of peripheral obstructive arterial disease. (http://www.jvascbr.com.br/vol4_n4_supl4.pdf)

G) Metabolic syndrome: diagnosed when a patient has at least 3 of the following 5 conditions:

- Fasting glucose ≥100 mg/dL (or receiving drug therapy for hyperglycemia)
- Blood pressure ≥130 or ≥ 85 mm Hg (or receiving drug therapy for hypertension)
- Triglycerides ≥150 mg/dL (or receiving drug therapy for hypertriglyceridemia)
- HDL-C <40 mg/dL in men or <50 mg/dL in women
- Waist circumference ≥102 cm in men or ≥88 cm in women

**Supplementary Table 1.** Characteristics of the subjects with stenoses in either cervical *or* intracranial arteries (Group_ExtraorIntra_), and in subjects with at least one cervical *and* one intracranial artery (Group_Extra&Intra_).

| **Characteristic** | **Group_Extra&Intra_ (n=67)** | **Group_ExtraorIntra_**  **(n=13)** | | ***p*-value** |  |
| --- | --- | --- | --- | --- | --- |
| **Age (years)** | 67.5±7.5 | 64.3±7.8 | | 0.175^b^ |  |
| **Education (years)** | 8.5±4.3 | 6.5±4.8 | | 0.174^b^ |  |
| **Male sex (%)** | 61.5 | 68.7 | | 0.616^a^ |  |
| **Ethnic group (%)**  **Black**  **White**  **Asian** | 30.8  61.5  7.7 | 44.8  52.2  3 | 0.538^d^ | |  |
| **Hypertension (%)** | 92.3 | 86.6 | 0.815^a^ | |  |
| **Diabetes (%)** | 53.8 | 43.3 | 0.484^a^ | |  |
| **Hyperlipidemia (%)** | 100 | 100 | 1.000^d^ | |  |
| **Family history of stroke (%)** | 61.5 | 49.3 | 0.417^a^ | |  |
| **Pooled Cohort Equations risk (%)** | 23.5±15.9 | 23.4±14.6 | 0.502^c^ | |  |
| **Smoking (%)** | 7.7 | 16.4 | 0.420^a^ | |  |
| **Ankle-brachial Index < 0.9 (%)** | 25 | 29.8 | 0.733^a^ | |  |
| **Metabolic Syndrome (%)** | 53.8 | 46.3 | 0.617^a^ | |  |
| **Antiplatelet agents (%)** | 84.6 | 94 | 0.238^a^ | |  |
| **Statins (%)** | 100 | 97 | 0.528^a^ | |  |
| **Anti-diabetic medications (%)** | 30.8 | 28.4 | 0.860^a^ | |  |

Means ± standard deviations are given.

^a^Chi-square test; ^b^ Student´s t-test; ^c^Mann-Whitney test; ^d^Likelihood test; ^e^Fisher´s test.

**Supplementary Table 2.** Multivariate analyses with PCE without statin (Model 2).

| **CAC** ≥ **100^a^** |  |  |
| --- | --- | --- |
| **Model 2** | **OR (CI)** | **P-value** |
| PCE without statin | 1.025 (1.002 – 1.049) | **0.035** |
| Group_athero_ | 1.754 (0.781 - 3.942) | 0.174 |
| **CAC > 0^b^** | | |
| **Model 2** | **OR (CI)** | **P-value** |
| PCE without statin  Group_athero_ | 1.029 (0.997 – 1.061)  4.203 (1.72 – 10.271) | 0.074  **0.002** |
| **Log (CAC +1)^c^** |  |  |
| **Model 2** | **Coefficient (CI)** | **P-value** |
| PCE without statin  Group_athero_ | 0.039 (0.013-0.065)  1.006 (0.084-1.928) | **0.004**  **0.033** |

^a^Multiple logistic regression: dependent variables, presence of coronary calcium scores (CAC) ≥ 100; Independent variables, scores in pooled cohort equations without statin and group (Group_athero_ or Group_control_).

^b^Multiple logistic regression: dependent variables, presence of coronary calcium scores (CAC) ≥ 0 Independent variables, scores in pooled cohort equations and group (Group_athero_ or Group_control_).

^c^Linear regression: dependent variable, logarithm of sum (absolute coronary calcium scores + 1). Independent variables, scores in pooled cohort equations and group (Group_athero_ or Group_control_).

OR,odds ratio. CI, conﬁdence interval. CAC, coronary artery calciﬁcation scores. PCE, *pooled cohort equations.*
